# Inhibition of USP7 activity selectively eliminates senescent cells in part via restoration of p53 activity

**DOI:** 10.1111/acel.13117

**Published:** 2020-02-16

**Authors:** Yonghan He, Wen Li, Dongwen Lv, Xin Zhang, Xuan Zhang, Yuma T. Ortiz, Vivekananda Budamagunta, Judith Campisi, Guangrong Zheng, Daohong Zhou

**Affiliations:** ^1^ Department of Pharmacodynamics College of Pharmacy University of Florida Gainesville FL USA; ^2^ Department of Medicinal Chemistry College of Pharmacy University of Florida Gainesville FL USA; ^3^ The Buck Institute for Research on Aging Novato CA USA; ^4^ Lawrence Berkeley National Laboratory Berkeley CA USA

**Keywords:** apoptosis, MDM2, p53, Senescence, senolytics, USP7

## Abstract

The accumulation of senescent cells (SnCs) is a causal factor of various age‐related diseases as well as some of the side effects of chemotherapy. Pharmacological elimination of SnCs (senolysis) has the potential to be developed into novel therapeutic strategies to treat these diseases and pathological conditions. Here we show that ubiquitin‐specific peptidase 7 (USP7) is a novel target for senolysis because inhibition of USP7 with an inhibitor or genetic depletion of *USP7* by RNA interference induces apoptosis selectively in SnCs. The senolytic activity of USP7 inhibitors is likely attributable in part to the promotion of the human homolog of mouse double minute 2 (MDM2) ubiquitination and degradation by the ubiquitin–proteasome system. This degradation increases the levels of p53, which in turn induces the pro‐apoptotic proteins PUMA, NOXA, and FAS and inhibits the interaction of BCL‐XL and BAK to selectively induce apoptosis in SnCs. Further, we show that treatment with a USP7 inhibitor can effectively eliminate SnCs and suppress the senescence‐associated secretory phenotype (SASP) induced by doxorubicin in mice. These findings suggest that small molecule USP7 inhibitors are novel senolytics that can be exploited to reduce chemotherapy‐induced toxicities and treat age‐related diseases.

## Graphical abstract

Some senescent cells (SnCs) are resistant to apoptosis in part due to down‐regulation of p53. Inhibition of USP7 can destabilize MDM2 (an E3 ligase targeting p53 for degradation) by promoting MDM2 ubiquitination and proteasome degradation, which in turn up‐regulates p53 and selectively induces apoptosis in SnCs by induction of the pro‐apoptotic proteins PUMA, NOXA and FAS and inhibition of the interaction of BCL‐XL and BAK. These findings suggest that USP7 inhibitors are novel senolytics.

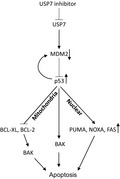

## 1 INTRODUCTION

Cellular senescence is a phenomenon by which replication‐competent cells cease to divide after extensive replication or exposure to stress (Hayflick et al., 1965; Childs et al., 2017). Although cellular senescence is an important tumor‐suppressive mechanism, emerging evidence demonstrates that the accumulation of senescent cells (SnCs) with age and after genotoxic or cytotoxic cancer therapy can lead to various age‐related diseases and pathological conditions (Demaria et al., 2017; Naylor, Baker, & Deursen, 2013). SnCs exert their deleterious effects in part via a senescence‐associated secretory phenotype (SASP) (Tchkonia, Zhu, Deursen, Campisi, & Kirkland, 2013). Therefore, selectively clearing SnCs and suppressing the SASP have emerged as attractive therapeutic strategies to extend health span (Baker et al., 2016), treat age‐related diseases (Baker et al., 2011; Childs et al., 2016; Jeon et al., 2017), and ameliorate chemotherapy‐induced toxicity (Demaria et al., 2017; He et al., 2019). However, SnCs are resistant to the induction of programmed cell death (apoptosis) (Marcotte, Lacelle, & Wang, 2004). The selective removal of SnCs depends on identifying their Achilles’ heels, which can be targeted to selectively kill SnCs. Several senolytic targets have been identified, resulting in the discovery of a series of senolytic agents that can selectively kill SnCs in culture and effectively remove SnCs in mice (Chang et al., 2016; Childs et al., 2017; Zhu et al., 2016). Unfortunately, some of these agents exhibit toxicities that may prevent their safe use in the clinic, particularly for systemic therapy. For example, navitoclax, a selective BCL‐2/XL dual inhibitor, is a potent senolytic agent but can induce thrombocytopenia, an on‐target and dose‐limiting toxicity that has prevented its FDA‐approval. Therefore, further studies are needed to identify new senolytic targets that can be exploited for the development of safer senolytic agents.

p53 is a well‐known tumor suppressor that acts as a double‐edged sword in regulating cellular senescence, aging, and cancer (Johmura & Nakanishi, 2016; Wu & Prives, 2018). Levels and activities of p53 increase when cells enter a presenescent state upon activation of the DNA damage response (DDR), which plays an important role in the initiation of cellular senescence. However, the levels of p53 decline in some cells after they become senescent, sometimes to a level below the basal levels of p53 in non‐SnCs. This phenomenon was observed in keratinocytes (Kim et al., 2015), some human fibroblast cell lines (Johmura et al., 2016; Sisoula, Trachana, Patterson, & Gonos, 2011), and human prostate epithelial and uroepithelial cells (Schwarze, Shi, Fu, Watson, & Jarrard, 2001). These observations suggest that a sustained upregulation of p53 may not be required for the maintenance of senescence in some SnCs, probably because they express a high level of p16 which provides another barrier to prevent the escape of senescence (Beauséjour et al., 2003). However, not all cells reduce their p53 expression when they become senescent (Rufini, Tucci, Celardo, & Melino, 2013). In addition, even though the levels of p53 return to the nearly basal levels in some SnCs after the acute DDR, a functional fraction of p53 may remain associated with chromatin to mediate the maintenance of senescence (Kirschner et al., 2015), particularly in cells expressing low levels of p16 (Beauséjour et al., 2003)*.*

The downregulation of p53 after some cells become senescent may be in part attributable to the upregulation of C‐terminus of Hsp70‐interacting protein (CHIP) (Sisoula et al., 2011) and SCF^Fbxo22^ (Johmura et al., 2016) E3 ligases, which promote p53 ubiquitination and proteasome degradation. SCF^Fbxo22^‐mediated p53 downregulation may play an important role for the expression of SASP in these SnCs, because the expression of SASP depends on the activation of p38‐MAPK and NF‐κB; and p53 can suppress SASP via inhibiting p38‐MAPK activity and competing with NF‐κB for transcriptional cofactors (Johmura et al., 2016). In addition, the reduction of p53 in these SnCs may protect them from apoptosis and contribute to the accumulation of SnCs and higher prevalence of cancer during aging because p53 is one of the most important apoptosis determinants, which can act through transcription‐dependent and transcription‐independent mechanisms (Fridman & Lowe, 2003). This suggestion is in agreement with the finding that several tissues in aged mice showed reduced p53 activity (Feng et al., 2007) and accumulation of SnCs (Baker et al., 2016). Therefore, restoration of p53 activity has the potential to eliminate these SnCs which downregulate their expression of p53 after they become senescent by inducing apoptosis. This hypothesis is supported by the finding that increasing p53 activity by disrupting its interaction with FOXO4 using an interfering peptide selectively induced apoptosis in some cultured SnCs and effectively cleared SnCs in fast aging *Xpd* ^TTD/TTD^ and naturally aged mice (Baar et al., 2017). However, there remain challenges using a peptide as a therapeutics and, again, not all SnCs use the same apoptosis‐avoiding mechanism. Thus, identifying new strategies to selectively kill SnCs by activating p53 will increase possibilities for extending health span and treating age‐related diseases and chemotherapy‐induced side effects.

p53 can also be activated by inhibiting the interaction between MDM2 and p53. The levels and activities of p53 are strongly regulated primarily by post‐transcriptional mechanisms, including MDM2‐mediated ubiquitination and proteasome degradation (Kruse & Gu, 2009). Inhibiting the interaction between MDM2 and p53 can increase p53 stability and activity (Moll & Petrenko, 2003). Indeed, recent findings show that UBX0101, an inhibitor of MDM2, selectively killed some SnCs in culture and effectively cleared them in mice with post‐traumatic osteoarthritis (Jeon et al., 2017). Similar to other senolytics derived from anticancer targeted agents, MDM2 inhibitors can cause hematopoietic suppression and gastrointestinal toxicity (Tisato, Voltan, Gonelli, Secchiero, & Zauli, 2017). It has yet to be determined whether these adverse effects are on‐target toxicities or off‐target side effects. Therefore, MDM2 inhibitors may be best for clearing SnCs to treat age‐related diseases such as osteoarthritis via local administration. It is thus important to find alternative strategies to activate p53 without causing severe toxicity but capable of effectively clearing SnCs systemically.

USP7 plays an important role in regulating p53 activity by deubiquitinating MDM2 to protect it from degradation by the ubiquitin–proteasome system (UPS). Thus, USP7 inhibition can stabilize p53 by promoting MDM2 auto‐ubiquitination and degradation (Li, Brooks, Kon, & Gu, 2004). Inhibition of USP7 has been proven effective in killing various cancer cells, in part by activating p53 and inducing apoptosis (Chauhan et al., 2012; Fan et al., 2013; Tavana et al., 2016). Unlike MDM2 inhibitors, USP7 inhibitors were well tolerated in mice at doses that effectively inhibited tumor growth (Chauhan et al., 2012; Fan et al., 2013; Tavana et al., 2016), suggesting that pharmacological inhibition of USP7 may be safer than inhibition of MDM2. Therefore, we examined whether USP7 is a novel senolytic target and whether inhibition of USP7 by a small molecule can selectively kill some SnCs in culture and clear the cells in vivo. Our results show that inhibition of USP7 by a small molecule or genetic depletion can selectively induce apoptosis in several types of SnCs at least in part via restoring p53 activity, which in turn induces the pro‐apoptotic proteins PUMA, NOXA, and FAS and inhibits the interaction of BCL‐XL and BAK because SnCs are more sensitive to the perturbation of mitochondrial apoptotic pathways than non‐SnCs (Chang et al., 2016; Yosef et al., 2016; Zhu et al., 2017). Furthermore, we show that treatment with a USP7 inhibitor can effectively eliminate SnCs and suppress the SASP induced by doxorubicin in mice without causing changes in blood cell counts and loss of body weight. These findings suggest that small molecule USP7 inhibitors are novel senolytics that can be exploited to reduce chemotherapy‐induced toxicities and treat age‐related diseases.

## 2 RESULTS

### 2.1 Inhibiting USP7 activity reduces MDM2 expression, increases p53 expression, and induces apoptosis selectively in SnCs

**Figure 1:**
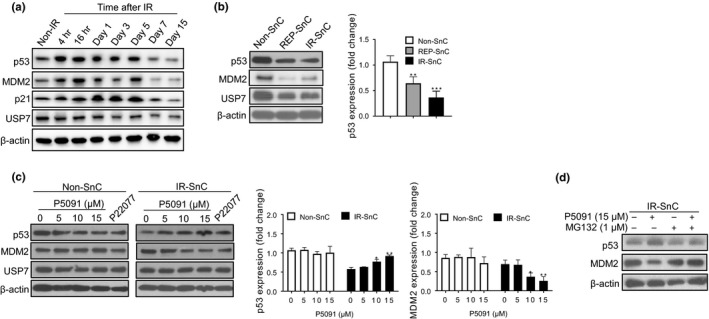
Inhibition of USP7 activity selectively downregulates MDM2 expression and partially restores p53 expression in senescent cells (SnCs). (a) Western blotting analysis of p53, MDM2 p21, and USP7 protein levels in nonradiated (non‐IR) and radiated WI‐38 fibroblasts at indicated time points. Representative images of Western blots are shown. (b) Western blotting analysis of p53, MDM2, and USP7 protein levels in nonsenescent (non‐SnC) and senescent (SnC) WI‐38 fibroblasts induced to senescence by replicative exhaustion (REP‐SnC) or ionizing radiation (IR‐SnC). Representative images of Western blots are shown in the left panel, and fold changes in p53 expression are presented in the right panel as mean ± *SEM* of 3 independent experiments with non‐SnC values set at 1. **p < .01 and ***p < .001. (c) Effect of USP7 inhibition by P5091 or P22077 on p53, MDM2 and USP7 protein levels in non‐SnC and IR‐SnC WI‐38 cells after the cells were treated with P5091 or P22077 (15 µM) for 72 hr. Representative images of Western blots are shown in the left panel, and fold changes in p53 and MDM2 levels relative to vehicle‐treated non‐SnC are presented in the right panel as mean ± *SEM* of 4 independent experiments. *p < .05 and **p < .01. (d) Proteasome inhibition by MG132 abrogated the effects of P5091 on MDM2 and p53 protein levels in IR‐SnCs. IR‐SnCs were pretreated with MG132 for 1 hr and then with P5091 for 72 hr. Representative images of Western blots are shown

As shown previously (Sisoula et al., 2011), basal levels of p53 in nonsenescent WI‐38 human fibroblasts were relatively low, but increased dramatically after exposure to ionizing radiation (IR) (Figure 1a). However, when the cells became senescent after exposure to IR, they expressed a significantly lower basal level of p53 compared to WI‐38 non‐SnCs (Figure 1a, b and Figure S1a, b). Replicative senescent WI‐38 cells also expressed a lower level of p53 than non‐SnCs (Figure 1b). Moreover, this phenomenon is not specific to WI‐38 cells because IMR‐90 fibroblasts, renal epithelial cells (RECs), and human umbilical vein endothelial cells (HUVECs) also expressed a significantly lower basal level of p53 when they became senescent (Figure S1c). However, the reduced basal levels of p53 in WI‐38 SnCs were unlikely attributable to the increased expression of MDM2 (Figure 1a, b).

To determine whether USP7 inhibition can selectively kill some SnCs via the MDM2‐p53 pathway, we examined the effect of USP7 inhibition on MDM2 and p53 levels in WI‐38 non‐SnCs and IR‐induced SnCs after treatment with different concentrations of P5091, a widely used USP7 inhibitor (Chauhan et al., 2012). P5091 downregulated MDM2 and upregulated p53 in WI‐38 SnCs but not in WI‐38 non‐SnCs (Figure 1c). We observed similar findings when these cells were treated with another USP7 inhibitor, P22077, and in IMR90 cells treated with P5091 (Figure 1c and Figure S1d). However, p53 levels in P5091‐treated WI‐38 SnCs remained slightly lower than the basal level of p53 in untreated non‐SnCs, suggesting that USP7 inhibition only partially restored the basal levels of p53 in WI‐38 SnCs and did not dramatically increase p53 expression as seen in WI‐38 cells after exposure to IR (Figure 1a, c). The changes in MDM2 and p53 expression in SnCs induced by USP7 inhibition were abrogated by suppressing proteasome activity using the inhibitor MG132 (Figure 1d). Thus, the changes in MDM2 and p53 expression in SnCs induced by USP7 inhibition relied on protein degradation by proteasomes.

**Figure 2:**
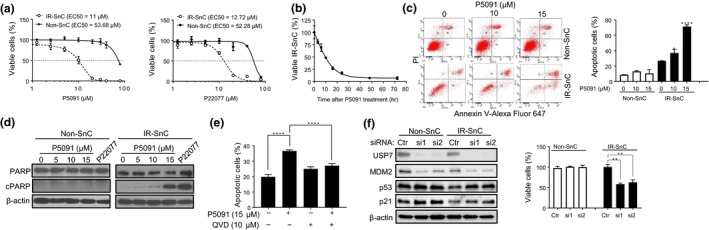
Inhibition of USP7 activity selectively induces apoptosis in SnCs. (a) Effect of USP7 inhibitors on cell viability of non‐SnC and IR‐SnC WI‐38 cells. Viability was determined after cells were treated with the indicated concentrations of P5091 or P22077 for 72 hr. Data are presented as mean ± *SEM* (*n* = 3). (b) P5091 induces cell death in IR‐SnC in a time‐dependent manner. IR‐SnC WI‐38 cells were treated with 15 µM P5091, and cell viability was determined at indicated time points. Data are presented as mean ± *SEM* (*n* = 4). (c) P5091 selectively induces apoptosis in IR‐SnC in a dose‐dependent manner. Apoptosis was determined after cells were treated with P5091 for 72 hr by Annexin V‐Alexa Fluor 647 and PI staining and flow cytometry. Representative flow cytometric plots are shown in the left panel, and percentages of apoptotic cells (Annexin V^+^ and Annexin V^+^/PI^+^ cells) are presented in the right panel as mean ± *SEM* of 3 independent experiments. *p < .05 and ****p < .0001. (d) Representative images of Western blots of PARP and cleaved PARP (cPARP) in non‐SnC and IR‐SnC WI‐38 cells 72 hr after treatment with P5091. (e) Percentages of apoptotic cells in IR‐SnC WI‐38 cells after pretreatment with the pan‐caspase inhibitor QVD for 4 hr, and then with or without P5091 for 24 hr. Data are presented as mean ± *SEM* (*n* = 3). ****p < .0001. (f) Knockdown of USP7 expression by siRNA reduces MDM2, upregulates p53, and induces apoptosis in IR‐SnCs. USP7, p53, and MDM2 protein levels were determined after transfection with control siRNA (Ctr), USP7 siRNA‐1 (si1), or USP7 siRNA‐2 (si2) for 48 hr. Cell viability in non‐SnC and IR‐SnC WI‐38 cells was determined 4 d after transfection. Representative images of Western blots are shown in the left panel, and percentages of viable cells are presented in the right panel as mean ± *SEM* of 3 independent experiments. **p < .01

Next, we examined the effect of USP7 inhibition on cell viability. IR‐induced WI‐38 SnCs were more sensitive to P5091 and P22077 than non‐SnCs (Figure 2a). Similar findings were observed in replicatively senescent WI‐38 and IMR‐90 fibroblasts, RECs, HUVECs, and human preadipocytes (PA) induced to senesce by IR or extensive replication (Figure S2a–e). The loss of SnC viability after incubation with P5091 occurred very rapidly (Figure 2b) and was primarily attributable to apoptosis because SnCs treated with P5091 showed significant increases in Annexin V staining (Figure 2c) and cleavage of poly(ADP‐ribose) polymerase (PARP) (Figure 2d). Further, the pan‐caspase inhibitor QVD abrogated the effect of P5091 on the viability of SnCs (Figure 2e).

To validate the specificity of the effects of USP7 inhibitors on SnCs, we transfected WI‐38 non‐SnC and SnC cells induced by IR with control or USP7 small interfering RNA (siRNA) to deplete USP7. The USP7 siRNA effectively reduced the expression of USP7 in both cells (Figure 2f), resulting in a significant reduction in MDM2 expression. However, consistent with the effects of the USP7 inhibitor, USP7 depletion increased p53 expression selectively in SnCs and reduced their viability while having no significant effects on non‐SnCs (Figure 2f). Furthermore, USP7 depletion by siRNA selectively reduced the viability of replicatively senescent WI‐38, and IR‐induced or replicatively senescent IMR90 cells (Figure S2f and g). These results confirm that USP7 is a novel senolytic target and inhibition of USP7 activity can selectively kill SnCs, probably in part by destabilizing MDM2 and upregulating p53.

### 2.2 Inhibition of USP7 activity induces SnC apoptosis in part via a p53‐dependent manner

**Figure 3:**
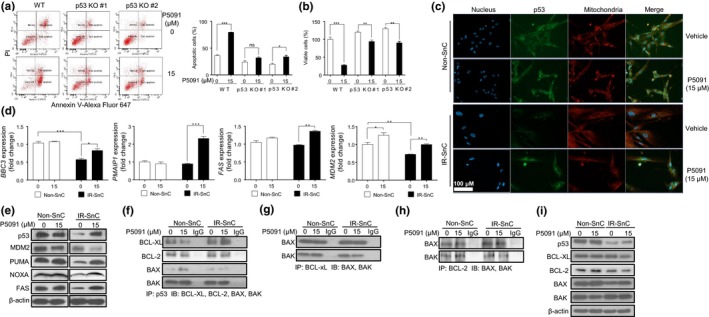
Inhibition of USP7 activity selectively induces apoptosis in SnCs partially through a p53‐dependent manner. (a) p53 knockout (p53 KO) attenuates P5091‐induced apoptosis in IR‐SnC WI‐38 cells. Apoptosis in wild‐type (WT) and p53 KO IR‐SnC WI‐38 cells was determined after treatment with vehicle or P5091 (15 µM) for 72 hr by Annexin V‐Alexa Fluor 647 and PI staining and flow cytometry. Representative flow cytometric plots are shown in the left panel, and percentages of apoptotic cells (Annexin V^+^ and Annexin V^+^/PI^+^ cells) are presented in the right panel as mean ± *SEM* of 3 independent experiments. *p < .05 and ***p < .001. (b) p53 knockout (p53 KO) attenuates the senolytic effect of P5091 on IR‐SnC WI‐38 cells. Cell viability in wild‐type (WT) and p53 KO IR‐SnC was determined after they were treated with vehicle or P5091 (15 µM) for 72 hr by PI staining using flow cytometry. Percentages of viable cells (PI^‐^ cells) are presented as mean ± *SEM* (*n* = 4). **p < .01 and ***p < .001. (c) Representative images of p53 immunofluorescent staining (green) in non‐SnC and IR‐SnC WI‐38 cells after the cells were treated with or without P5091 for 16 hr. The mitochondria and nucleus were stained with MitoTracker Red CMXRos (red) and DAPI (blue), respectively. (d) *BBC3, PMAIP1, FAS,* and *MDM2* mRNA levels in non‐SnC and IR‐SnC WI‐38 cells after treatment with P5091 for 9 hr were measured by quantitative PCR (qPCR). Data are presented as mean ± *SEM* (*n* = 3) of fold changes. *p < .05, ***p < .001, and ****p < .0001. (e) Representative images of Western blots of p53, MDM2, PUMA, NOXA, and FAS in non‐SnC and IR‐SnC WI‐38 cells after treatment with or without P5091 for 72 hr. (f) Analysis of the interaction of p53 with BCL‐XL, BCL‐2, BAK, and BAX in non‐SnC and IR‐SnC WI‐38 cells after treatment with vehicle or P5091 for 16 hr by immunoprecipitation (IP) and immunoblotting (IB). (g) Co‐IP of BCL‐XL with BAK and BAX in non‐SnC and IR‐SnC WI‐38 cells with or without P5091 treatment for 16 hr. (h) Co‐IP of BCL‐2 with BAK and BAX in non‐SnC and IR‐SnC WI‐38 cells with or without P5091 treatment for 16 hr. (i) Levels of p53, BCL‐XL, BCL‐2, BAX, and BAK in the lysates used for the IP experiments presented in (f‐h)

To determine whether p53 is required for mediating USP7 inhibition‐induced apoptosis in SnCs, we generated p53 knockout WI‐38 cells using CRISPR/cas9 technology (Figure S3a). Knockout p53 had no significant effect on the induction of senescence by IR because both wild‐type (WT) and p53 knockout WI‐38 cells became permanently growth arrested (i.e., inability to incorporate bromodeoxyuridine), expressed increased levels of p16 and p21 and stained positive for senescence‐associated β‐galactosidase (SA‐β‐gal) after exposure to IR (Figure S3b–d). These results suggest that p53 is dispensable for the induction of cellular senescence by IR, as reported (Nair, Bagheri, & Saini, 2015). However, senescent p53 knockout cells were resistant to apoptosis and cell death caused by USP7 inhibition with P5091 (Figure 3a, b). In contrast, wild‐type WI‐38 SnCs underwent apoptosis after treatment with P5091. This result confirms that USP7 inhibition at least in part depends on p53 for the selective induction of SnC apoptosis.

p53 is a transcriptional factor that induces apoptosis primarily by inducing the expression of pro‐apoptotic genes, including *BBC3* (encoding PUMA), *PMAIP1* (encoding NOXA), and *FAS* (Fridman & Lowe, 2003). In addition, p53 can also induce apoptosis in a transcription‐independent manner by translocating into mitochondria to interfere with the interaction between anti‐apoptotic BCL‐family proteins and pro‐apoptotic proteins (Speidel, 2010). Therefore, we performed p53 immunofluorescent staining to determine p53 distribution in non‐SnCs and SnCs with or without P5091 treatment (Figure 3c and Figure S3e). The specificity of the staining was validated using p53 knockout cells (Figure S3e). As expected, p53 staining was significantly lower in SnCs than non‐SnCs, which was restored after P5091 treatment. In P5091‐treated SnCs, some p53 staining was located in nuclei but the majority of the staining appeared to be in cytoplasm in association with mitochondria (Figure 3c and Figure S3e). These findings were confirmed by Western blotting analysis using SnC cytoplasmic, mitochondrial, and nuclear protein lysates (Figure S3f).

To determine whether p53 mediates USP7 inhibition‐induced SnC apoptosis by upregulating pro‐apoptotic genes, we compared *BBC3*, *PMAIP1,* and *FAS* mRNA levels in non‐SnCs and IR‐induced SnCs with or without P5091 treatment. Untreated SnCs expressed significantly lower levels of *BBC3* mRNA than non‐SnCs. USP7 inhibition had no significant effect on the levels of *BBC3*, *PMAIP1,* and *FAS* mRNA in non‐SnCs, but slightly elevated *BBC3* mRNA in SnCs (Figure 3d). Although the expression of *PMAIP1* and *FAS* mRNA was not reduced in SnCs, their expression was selectively elevated in SnCs after P5091 treatment. A similar change in SnC expression of PUMA, NOXA, and FAS at the protein level was observed by Western blotting analysis (Figure 3e). Moreover, these changes correlated with the levels of p53, indicating that USP7 inhibition can partially restore the expression of p53 and its downstream pro‐apoptotic proteins in SnCs. These findings suggest that increased p53 transcriptional activity may be in part responsible for the induction of SnC apoptosis by USP7 inhibition.

In contrast, P5091 increased the expression of *MDM2* mRNA but reduced the expression of MDM2 protein in SnCs (Figure 3d, e), which was abrogated by the pretreatment of the cells with the proteasome inhibitor MG132 (Figure 1c). These findings are in agreement with our suggestion that USP7 inhibition upregulates p53 expression at least in part via promoting MDM2 proteasome degradation. However, the expression of p21 mRNA in SnCs was elevated in comparison with non‐SnCs and its expression was not affected by P5091 treatment (Figure S3g). These findings suggest that p21 mRNA expression in SnCs can be regulated in a p53‐independent manner, which is in agreement with the findings reported previously (Aliouat‐Denis et al., 2005).

Next, we examined whether USP7 inhibition can promote p53 interaction with mitochondrial anti‐apoptotic BCL‐family proteins to release pro‐apoptotic proteins for the induction of SnC apoptosis by immunoprecipitation (Figure 3f‐i). p53 complexed with BAK, but to a lesser degree to BAX, in both non‐SnCs and SnCs, regardless of whether the cells were treated with P5091 (Figure 3f). Slightly more p53 complexed with BCL‐XL in SnCs than non‐SnCs without P5091 treatment. After P5091 treatment, the p53‐BCL‐XL interaction increased further in SnCs but decreased slightly in non‐SnCs. Importantly, P5091 selectively reduced the interaction between BCL‐XL and BAK in SnCs but not in non‐SnCs (Figure 3g). However, P5091 had no significant effect on the interaction between BCL‐2 and p53, nor on the interactions between BCL‐2 and BAX or BAK (Figure 3h and i).

These findings suggest that USP7 inhibition selectively induces SnC apoptosis at least in part by increasing p53 translocation to mitochondria and its interaction with BCL‐XL, since SnCs are more dependent on BCL‐2 family proteins for survival compared to non‐SnCs (Chang et al., 2016; Yosef et al., 2016; Zhu et al., 2016). In addition, we predict that USP7 inhibition may sensitize SnCs to apoptosis via BCL‐2/XL inhibition. Indeed, compared to non‐SnCs, SnCs have more mitochondria (Figure S4a) and higher levels of BCL‐XL and BCL‐2 (Figure S4b and c), and the combination of P5091 and ABT263, a BCL‐2/XL inhibitor, was more effective in killing SnCs than either agent alone (Figure S4d).

### 2.3 USP7 inhibition eliminates SnCs and reduces the SASP induced by doxorubicin in mice

**Figure 4:**
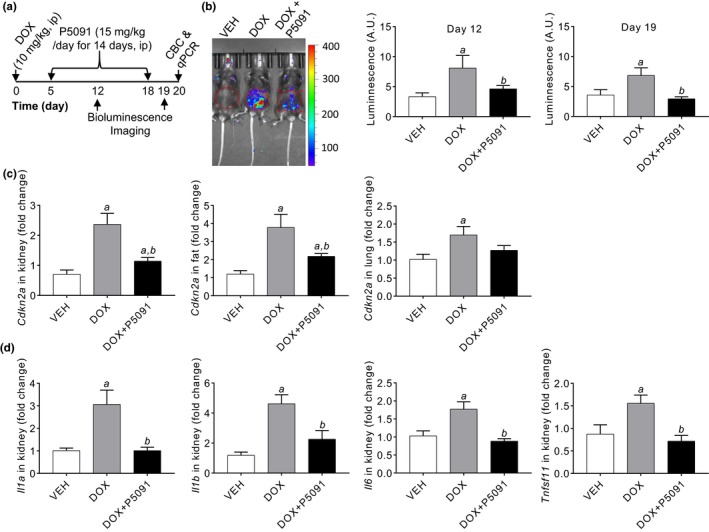
Treatment with the USP7 inhibitor P5091 effectively eliminates SnCs and reduces expression of senescence‐associated secretory phenotype (SASP) factors induced by doxorubicin (DOX) in mice. (a) Experimental design. CBC, complete blood count. (b) A representative bioluminescence image of p16‐3MR mice after treatment with vehicle (VEH), DOX or DOX plus P5091 is shown in the left panel and the relative intensities of luminescence on day 12 and 19 are presented in the right panel as mean ± *SEM* (*n* = 3 mice/group). (c) The levels of p16 mRNA in the kidney, inguinal fat, and lungs and (d) the levels of SASP factors *Il1α*, *Il1β*, *Il6,* and *Tnfsf11* mRNA in the kidney from vehicle (VEH), DOX,or DOX plus P5091‐treated mice were quantified by qPCR and are presented as mean ± *SEM* of fold changes in comparison with those from vehicle‐treated mice (*n* = 6). *a*, *p* < .05 and *b*, *p* < .05 versus. VEH and DOX group, respectively

We used p16‐3MR mice treated with doxorubicin (DOX) to determine whether USP7 inhibition could effectively eliminate SnCs in vivo (Figure 4a). Treatment with a chemotherapeutic drug such as DOX induces cellular senescence in mice, which contributes to chemotherapy‐induced toxicities in part due to the SASP (Demaria et al., 2017). As shown in our previous study (Demaria et al., 2017), p16‐3MR mice exhibited significant increases in SnC accumulation after DOX treatment based on bioluminescence imaging (Figure 4b), which was confirmed by analyzing *Cdkn2a* mRNA expression in the kidney, fat, and lungs using qPCR (Figure 4c). Furthermore, kidneys from DOX‐treated mice expressed increased levels of mRNAs encoding various SASP factors, including IL‐1α, IL‐1β, IL‐6, and RANKL compared to vehicle‐treated mice (Figure 4d). Treatment with the USP7 inhibitor P5091 not only almost completely eliminated SnCs but also abrogated DOX‐induced increase in the levels of *Il1α*, *Il1β*, *Il6,* and *Tnfsf11* mRNAs (Figure 4d). These findings demonstrate that P5091 effectively cleared SnCs induced by DOX in vivo. Importantly, mice treated with P5091 for 14 days exhibited no significant changes in body weight and no reduction in the number of white blood cells, red blood cells, platelets, and neutrophils and the level of hemoglobin (Figure S5). These results suggest that USP7 inhibitors could be safer than MDM2 inhibitors for senolysis.

## 3 DISCUSSION

Downregulation of p53 has been identified as one of the mechanisms by which some SnCs gain resistance to apoptosis (Chaturvedi, Qin, Stennett, Choubey, & Nickoloff, 2004). The downregulation is not due to an increased expression of MDM2 as seen in WI‐38 SnCs, but may be attributable to an increased expression of F‐Box Protein 22 (Fbxo22) and SCF^Fbxo22^‐KDM4A E3 ligase activity, which can induce p53 ubiquitination and degradation (Johmura et al., 2016). In addition, some cells can downregulate p53 expression at the transcriptional level after they become senescent (Kim et al., 2015). Here we report the discovery of a novel approach to selectively increase p53 expression and induce apoptosis in SnCs via USP7 inhibition. The mechanism by which USP7 inhibition increases p53 expression is likely attributable in part to the reduction of MDM2 expression as USP7 stabilizes MDM2 via deubiquitinating MDM2 (Li et al., 2004). This hypothesis is supported by our finding that proteasome inhibition with MG132 abrogated USP7 inhibition‐induced downregulation of MDM2 and upregulation of p53 in SnCs. Interestingly, USP7 inhibition had no significant effect on the expression of MDM2 and p53, nor did it significantly increase apoptosis in non‐SnCs. Thus, USP7 is a new senolytic target and USP7 inhibitors are novel senolytic agents at least for some SnCs which downregulate their expression of p53 after they become senescent.

However, USP7 has multiple targets (Pozhidaeva & Bezsonova, 2019) and p53 knockout only attenuated but did not abrogate USP7 inhibition‐induced SnC apoptosis. Therefore, it is possible that USP7 inhibition may induce SnC apoptosis not only via a p53‐dependent manner but also through a p53‐independent mechanism. It has been shown previously that USP7 inhibition can induce apoptosis in various cancer cells via restoration of PTEN nuclear pool (Carrà et al., 2017), inhibition of Wnt signaling (An et al., 2017), and induction of oxidative and endoplasmic reticulum stress (Lee et al., 2016). However, whether any of these mechanisms contribute to USP7 inhibition‐induced SnC apoptosis has yet to be determined.

The results from our studies also suggest that p53 may mediate USP7 inhibition‐induced SnC apoptosis via both transcriptional and post‐transcriptional mechanisms. First, we found that upregulation of p53 expression in SnCs after USP7 inhibition with P5091 treatment increased the expression of several pro‐apoptotic protein genes including *PUMA*, *NOXA,* and *FAS* mRNA. In addition, we showed that p53 induced by USP7 inhibition in SnCs localized partially to mitochondria, where it might induce SnC apoptosis by interacting with BCL‐XL and displacing BAK. This finding is in agreement with the observation that inhibiting the interaction between FOXO4 and p53 by a FOXO4‐D‐retro‐inverso (DRI) peptide selectively induced apoptosis in SnCs in a p53 transcriptional activity‐independent manner by promoting p53 translocation from the nuclei to the mitochondria (Baar et al., 2017). This mechanism may contribute to the selective induction of apoptosis in SnCs by USP7 inhibition because studies from our groups and others show that SnCs are more dependent on BCL‐XL for survival (Chang et al., 2016; Yosef et al., 2016; Zhu et al., 2016). Therefore, a combination treatment with the USP7 inhibitor P5091 and BCL‐XL inhibitor ABT263 could synergistically kill SnCs, as shown in our studies.

Using a small molecule USP7 inhibitor to selectively upregulate p53 and induce apoptosis in SnCs may be a good alternative to the use of FOXO4‐DRI and MDM2 small molecule inhibitors as the use of peptides as a therapeutic remains a challenge, and MDM2 inhibitors can be toxic to the hematopoietic and gastrointestinal systems (Tisato et al., 2017). Treatment of mice with the USP7 inhibitor P5091 effectively eliminated SnCs and reduced the expression of SASP factors induced by DOX without affecting body weight or blood cell counts. However, most known USP7 inhibitors, including P5091, are still in early development and not ready for extensive studies to examine senolytic activity or toxicity in vivo. With an increasing interest and effort in the development of USP7 inhibitors for cancer therapy (Chauhan et al., 2012; Fan et al., 2013; Tavana et al., 2016), more specific and potent USP7 inhibitors may become available in the near future. However, USP7 is expressed in many tissues and regulates other substrates that have different physiological functions (Zhou et al., 2018). Therefore, it remains to be determined whether USP7 inhibitors can be safely used to extend health span and treat age‐related diseases, particularly because older people are more susceptible to adverse drug effects than younger individuals. Since USP7 inhibitors are developed primarily for oncology (Zhou et al., 2018), a more viable approach to use USP7 inhibitors as senolytic agents may be to clear SnCs induced by chemotherapy because such cells can contribute to chemotherapy‐induced toxicity and promote tumor relapse and metastasis in part via the SASP. This suggestion is supported by our finding that treatment of mice with the USP7 inhibitor P5091 effectively eliminated SnCs and reduced the expression of SASP factors caused by DOX.

In conclusion, we reveal that USP7 is a novel senolytic target, and USP7 inhibition can selectively kill some SnCs with p53 downregulation in vitro and clear SnCs induced by chemotherapy in mice in part by destabilizing MDM2 to increase p53 expression. However, not all cells reduce their p53 expression when they become senescent (Rufini et al., 2013). It has yet to determine whether SnCs without p53 downregulation are also sensitive to USP7 inhibition‐induced apoptosis and whether USP7 inhibitors can be used as a broad‐spectrum senolytic agent.

## 4 EXPERIMENTAL PROCEDURES

### 4.1 Cell culture and induction of senescence

Human WI‐38 (catalog no. CCL‐75) and IMR‐90 (catalog no. CCL‐186) fibroblasts, HUVEC (catalog no. CRL‐1730), REC (catalog no. PCS‐400‐012, REC), and PA cells (catalog no. PCS‐210–010) were purchased from American Type Culture Collection (ATCC, Manassas, VA, USA). Cells were cultured as described (Li et al. 2019). We considered cells at low passage (WI‐38 and IMR‐90 < 25 passages; HUVEC and REC < 10 passages; PA < 4 passages) as non‐SnCs. These non‐SnCs used in our study were healthy and subconfluent. For example, WI‐38 non‐SnCs expressed low levels of p16 (CDKN2a) and p21 (CDKN1a) mRNA and were capable of proliferating and synthesizing DNA (positive for BrdU staining) (Figure S1a and b). Senescence induction was done as previously reported (Li et al., 2019).

### 4.2 p53 knockout by CRISPR/Cas9 genomic editing

Two different sgRNAs targeting human p53 were cloned into the lentiCRISPR v2 vector (a gift from Feng Zhang; Addgene plasmid # 52,961) (Sanjana, Shalem, & Zhang, 2014). Packaging 293T cells were transfected with sgRNAs or negative controls (nontargeting sgRNA‐NC) and helper vectors (pMD2.G and psPAX2; Addgene plasmid #s12259 and 12,260) using Lipofectamine 2000 reagent. Medium containing viral particles and 8 μg/mL polybrene was used to infect cells. Infected cells were selected in medium containing 2 μg/mL puromycin. Reagents used are provided in Table S1. The guide sequences are provided in Table S2.

### 4.3 USP7 gene silencing

Non‐SnCs and IR‐SnCs were transfected with a USP7 siRNA oligonucleotide duplex (siRNA ID: s15440 and s15441, Thermo Fisher Scientific, Waltham, MA, USA) using Lipofectamine RNAiMax (Table S1). Generally, siRNA and Lipofectamine RNAiMax were individually diluted in Opti‐MEM medium, gently mixed, incubated for 20 min at room temperature, and then added to cells at a final concentration of 50 nM siRNA. The effectiveness of siRNA knockdown was determined on days 2 and 5 after transfection by measuring the level of USP7 protein by Western blotting.

### 4.4 Cell viability assay and EC_50_ calculation

Cell viability was measured as previously described (Chang et al., 2016; Li et al., 2019), or by CellTiter‐Glo® Luminescent Cell Viability Assay kit (Table S1) as per the manufacturer's instructions. Dose responses were determined for each compound, and half‐maximal effective concentrations (EC_50_ values) were calculated using GraphPad Prism 6 software.

### 4.5 Apoptosis assay

Apoptosis assay was done as previously reported (Li et al., 2019). Reagents for this assay are provided in Table S1.

### 4.6 Western blotting

Western blotting was done as previously reported (Li et al., 2019). All regents and primary antibodies used are provided in Tables S1 and S3.

### 4.7 Cell fraction isolation and co‐immunoprecipitation

Cell fractions were isolated using the Cell Fractionation Kit with minor modifications. In order to concentrate mitochondrial and nuclear proteins, less volume of Buffer C (half of Buffer B) was used to re‐suspend the pellet after isolating cytoplasmic protein. GAPDH, Cox IV, and PARP were used as markers for cytoplasmic, mitochondrial, and nuclear fractions, respectively. For co‐immunoprecipitation (Co‐IP), total protein was extracted using the Pierce IP Lysis Buffer supplemented with 1% Protease Inhibitor Cocktail and 1% Phosphatase Inhibitor Cocktail. 500–1,000 µg protein was incubated with anti‐p53, anti‐BCL‐XL, BCL‐2, or nonspecific antibodies overnight. Antibodies were crossed‐linked to Pierce Protein A/G Magnetic Beads for 2 hr. After washing 3 times with IP lysis buffer, proteins were eluted with 50 µl IP lysis buffer and 50 µl loading buffer. The eluates were boiled and analyzed by Western blotting. All regents and antibodies are provided in Tables S1 and S3.

### 4.8 RNA extraction and real‐time PCR analysis

Total RNA extraction was done as previously reported (Li et al., 2019). Real‐time PCR was performed using specific Taqman probes (Table S4) and the TaqMan Fast Advanced Master Mix. All reagents and kits are provided in Table S1. All experiments were performed in 2–3 replicates. Cell data were normalized to *GAPDH,* and tissue data were normalized to *Hprt*.

### 4.9 Immunofluorescence

Non‐SnCs and IR‐SnCs were seeded in glass bottom microwell dishes, treated with 15 µM P5091 overnight, then incubated with 150 nM MitoTracker Red CMXRos in culture medium per the manufacturer's instructions. After blocking with 3% BSA, anti‐p53 antibody was added (1:500 dilution) and incubated at room temperature for 1 hr followed by incubation with anti‐mouse IgG‐Alexa Fluo488 (1:2000 dilution) for 30 min at room temperature. After removing antibodies, cells were rinsed with PBS 3 times. Nuclei were stained using 0.25 µg/ml DAPI for 5 min at room temperature. Cells were rinsed with PBS 3 times and mounted with PBS and 1% BSA. Cells were viewed and photographed immediately using an Axioplan microscope (Carl Zeiss Inc, Jena, Germany) equipped with a 100 W mercury light source. Images were processed using Image‐Pro Plus software (Media Cybernetics, Rockville, Maryland, USA). All regents and antibodies used are provided in Tables S1 and S3.

### 4.10 Senescence‐associated β‐galactosidase (SA‐β‐gal) assay and BrDU Staining

SA‐β‐gal activity was determined using the β‐Galactosidase Staining Kit (Table S1), and DNA synthesis was detected using the Click‐iT™ Plus EdU Alexa Fluor™ 488 Imaging Kit (Table S1) according to the manufacturer's instructions.

### 4.11 Mice

Young (2 months old) p16‐3MR mice were bred at the University of Florida (UF) animal facility certified by the Association for the Assessment and Accreditation of Laboratory Animal Care International (AAALAC). Mice were randomly assigned to 5 per cage, housed at the facility and received food and water ad libitum. Where indicated, p16‐3MR mice were injected i.p. once with 10 mg/kg DOX in PBS, then, 5 days later, given daily i.p. injections of vehicle (4% NMP, 3% Tween‐80 and 20% PEG400 in Milli‐Q water) or P5091 (15 mg/kg) for 14 days. Body weight was measured every two days. In vivo bioluminescence was performed as described (Chang et al., 2016). One day after the second bioluminescence imaging, blood samples were collected for complete blood count (CBC) analysis using a HEMAVET® analyzer (Drew Scientific, Inc., Oxford, CT, USA). Mice were euthanized by CO_2_ suffocation followed by cervical dislocation. Tissues were harvested and immediately frozen in liquid nitrogen and stored at −80°C until processing. The Institutional Animal Care and Use Committees of UF approved all experimental procedures used in this study.

### 4.12 Statistical analysis

Data are expressed as means ± *SEM* and analyzed by analysis of variance (ANOVA) using Graphpad Prism from GraphPad Software (San Diego, CA, USA). Post hoc comparisons were performed between group means, comparisons made using Newman–Keuls or Tukey's multiple comparisons test*.* Comparisons were made by Student's *t* test when comparing two experimental groups. *p* < .05 was considered significant.

## CONFLICT OF INTEREST

Y.H.H., X(in).Z., G.Z., and DZ filed a patent application for the use of USP7 inhibitors as anti‐aging agents. JC and DZ are co‐founders of, and GZ, JC and DZ are advisors to, Unity Biotechnology, which develops small molecule, senolytic drugs.

## AUTHOR CONTRIBUTIONS

Y.H.H designed and performed the majority of the experiments, analyzed the data, and wrote the manuscript; W.L., D. L, X(in).Z., X(uan).Z., Y.T.O., and V.B. assisted with some of the experiments; J.C., and G.Z. analyzed the data and revised the manuscript; D.Z. conceived the project, designed the experiments, analyzed the data, and wrote the manuscript.

## Data Availability

The data that support the findings of this study are available from the corresponding author upon reasonable request.
