# Supplementary material for: Inhibition of USP7 activity selectively eliminates senescent cells in part via restoration of p53 activity
Source: Aging Cell. 2020 Feb 16;19(3):e13117. doi: 10.1111/acel.13117 (PMC7059172; doi:10.1111/acel.13117)
Supplement: Supplementary file 1 [file ACEL-19-e13117-s001.pdf]

Supporting Information

He Y et al. Inhibition of USP7 activity selectively eliminates senescent cells in part via restoration of p53 activity

**Supplementary Item**	**Title**
Supplementary Figure 1	Comparison of non-senescent (non-SnCs) and senescent cells (SnCs)
Supplementary Figure 2	Inhibition of USP7 activity selectively kills SnCs in a cell-type independent manner
Supplementary Figure 3	p53 knockout did not affect the induction of senescence in WI-38 cells by IR
Supplementary Figure 4	Synergistic killing effect of P5091 and ABT263 on SnCs
Supplementary Figure 5	Treatment with P5091 does not cause observable toxicity in mice
Supplementary Table 1	Reagents and supplies used in *in vitro* and *in vivo* experiments
Supplementary Table 2	SgRNA sequence for p53 knockout by CRISPR/Cas9 genomic editing
Supplementary Table 3	Antibodies used for Western blotting analyses
Supplementary Table 4	Taqman gene expression probes used for qRT-PCR

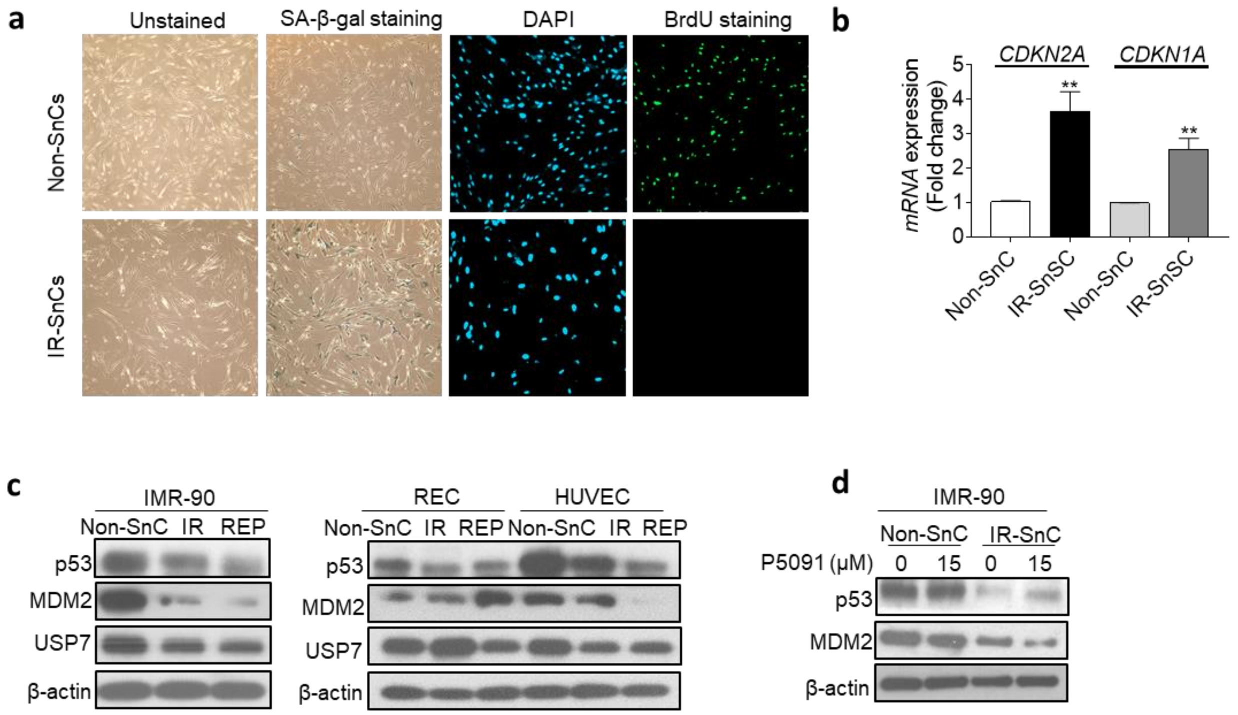

Supplementary Fig. 1. Comparison of non-senescent (non-SnC) and senescent cells (SnC). (**a**) Senescence-associated beta-galactosidase (SA-β-gal), DAPI (nucleus) and BrdU (DNA incorporation) staining in non-SnC and IR-SnC WI-38 cells. (**b**) Levels of *CDKN2A* (*p16*) and *CDKN1A* (*p21*) mRNA in non-SnC and IR-SnC WI-38 cells. **$p<0.01$ vs. non-SnC. (**c**) Expression of p53, MDM2 and USP7 in non-SnC and SnC IMR-90 fibroblast cells (left panel), renal epithelial cells (REC) and human umbilical vein endothelial cells (HUVEC) (right panel) induced by ionizing radiation (IR) or extensive replication (REP). (**d**) USP7 inhibition with P5091 selectively reduces MDM2 and increases p53 expression in IR-SnC IMR-90 cells.

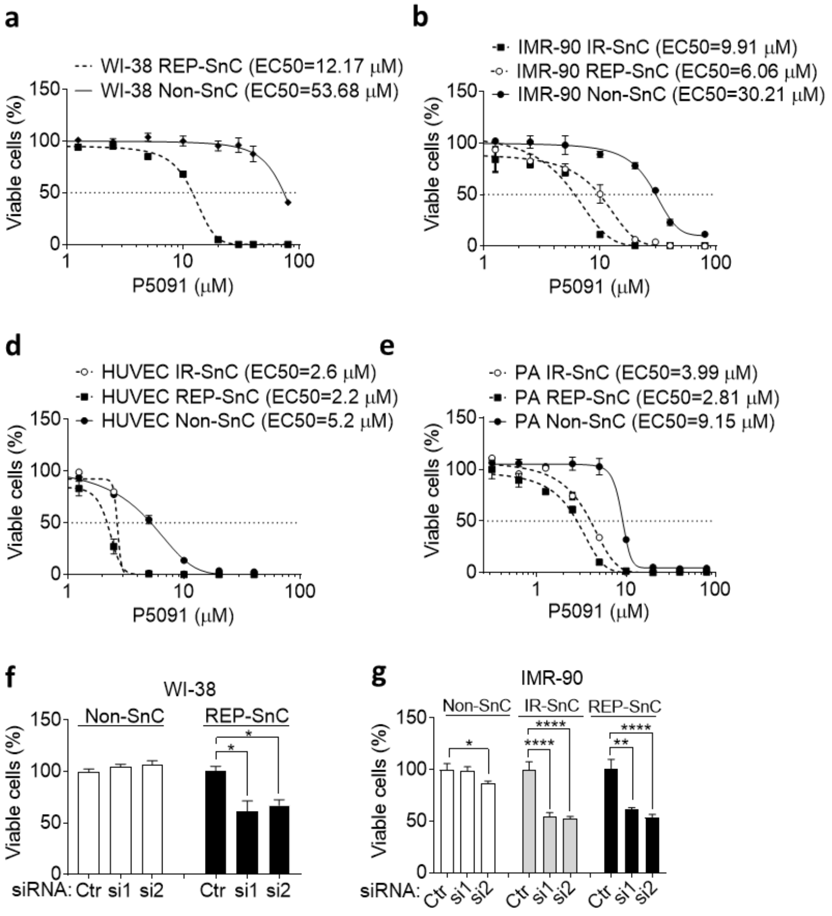

Supplementary Fig. 2. Inhibition of USP7 activity selectively kills SnCs in a cell-type independent manner. (**a-e**) Effect of USP7 inhibition with P5091 on cell viability in non-SnC, SnCs induced by IR (IR-SnC) or extensive replication (REP-SnC). WI-38, WI-38 fibroblast cells; IMR-90, IMR-90 fibroblast cells; REC, renal epithelial cells; HUVEC, human umbilical vein endothelial cells; and PA, pre-adipocytes. Cell viability was determined after they were treated with different concentrations of P5091 for 72 h (n = 3). (**f**) Effect of USP7 knockdown with siRNA on cell viability in non-SnC and REP-SnC WI-38 cells, and non-SnC, IR-SnC and REP-SnC IMR-90 cells. Ctr, control USP7 siRNA; si1, USP7 siRNA 1; and si2, USP7 siRNA 2. All the data are presented as mean ± SEM (n = 3). *p<0.05, **p<0.01, and ****p<0.0001 vs. Ctr.

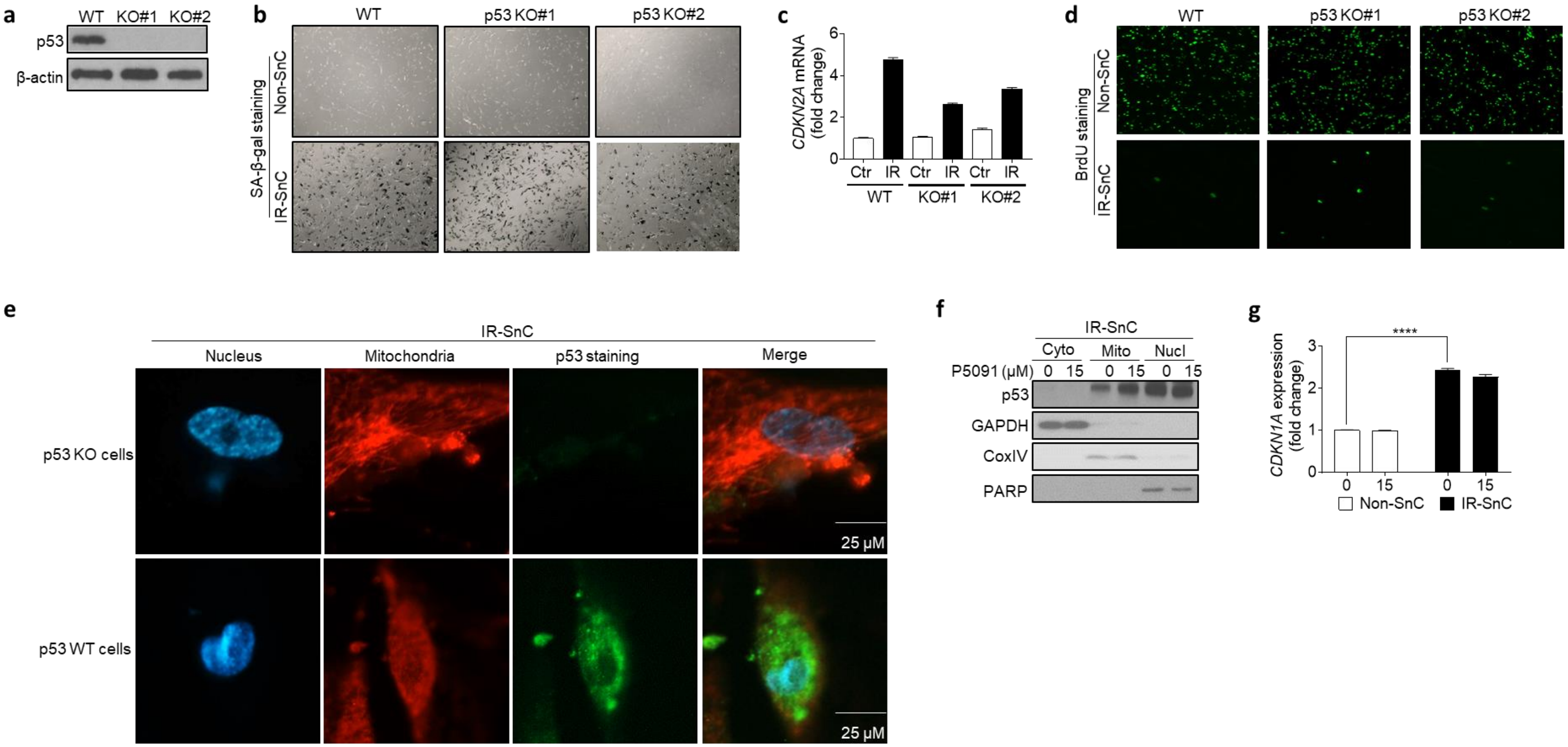
a
WT
KO#1
KO#2
p53
β-actin
b
WT
p53 KO#1
p53 KO#2
SA-β-gal staining
Non-SnC
IR-SnC
c
CDKN2A mRNA
(fold change)
0
2
4
6
Ctr
IR
Ctr
IR
Ctr
IR
WT
KO#1
KO#2
d
WT
p53 KO#1
p53 KO#2
BrdU staining
Non-SnC
IR-SnC
e
IR-SnC
Nucleus
Mitochondria
p53 staining
Merge
p53 KO cells
p53 WT cells
25 μM
25 μM
f
IR-SnC
Cyto
Mito
Nucl
P5091 (μM)
0
15
0
15
0
15
p53
GAPDH
CoxIV
PARP
g
CDKN1A expression
(fold change)
0
1
2
3
****
0
15
0
15
Non-SnC
IR-SnC

Supplementary Fig. 3. p53 knockout did not affect the induction of senescence in WI-38 cells by IR. (**a**) Confirmation of the efficiency of p53 knockout (KO) in WI-38 cells using the CRISPR/cas9 technology by Western blotting. WT, wild-type cells; KO#1, p53 sgRNA 1 generated p53 KO cells; and KO#2, p53 sgRNA 2 generated p53 KO cells. (**b**) Representative SA-β-gal staining images demonstrate that both WT and p53 KO WI-38 cells underwent senescence after exposure to IR. (**c**) Expression of *CDKN2A* (*p16*) mRNA in WT, p53 KO#1, and p53 KO#2 WI-38 cells before (Ctr) or after exposure to IR. (**d**) Representative BrdU staining images demonstrate that both WT and p53 KO WI-38 cells became permanently growth arrested after exposure to IR. (**e**) Representative images of p53 immunofluorescent staining (green) in WT and p53 KO IR-SnC WI-38 cells. The mitochondria and nucleus were stained with MitoTracker Red CMXRos (red) and DAPI (blue), respectively. (**f**) Representative images of Western blotting analysis of p53 expression in cytoplasm, mitochondria and nuclei in IR-SnC WI-38 cells after they were treated with vehicle or P5091 overnight. GAPDH, CoxIV and PARP were used to validate fraction purity. Cyto, cytoplasm; Mito, mitochondria; Nucl, nucleus. (**g**) Expression of *CDKN1A* (*p21*) mRNA in in non-SnC and IR-SnC WI-38 cells after treatment with P5091 for 9 h were measured by quantitative PCR (qPCR). Data are presented as mean ± SEM (n=3) of fold changes. ****$p<0.0001$.

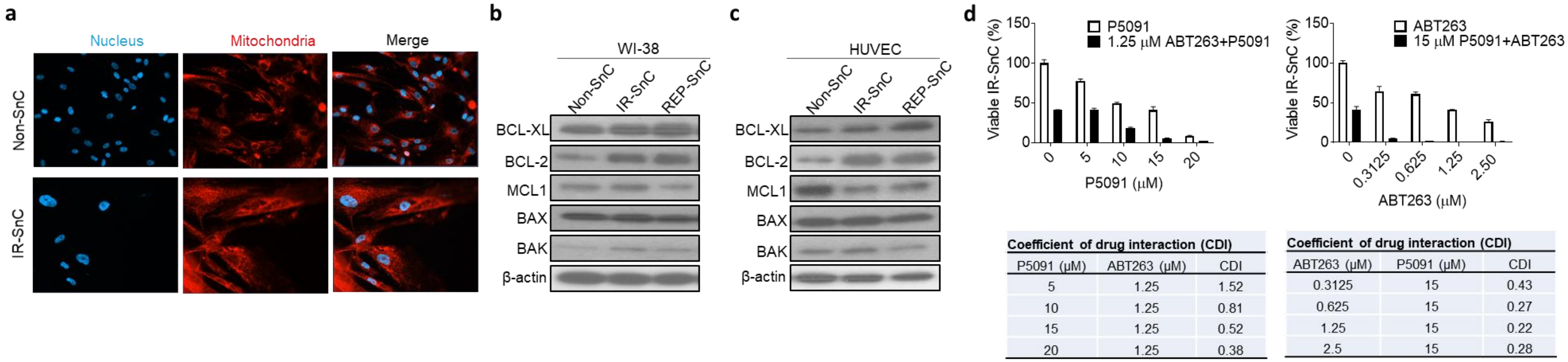

Coefficient of drug interaction (CDI)

P5091 (μM)	ABT263 (μM)	CDI
5	1.25	1.52
10	1.25	0.81
15	1.25	0.52
20	1.25	0.38

Coefficient of drug interaction (CDI)

ABT263 (μM)	P5091 (μM)	CDI
0.3125	15	0.43
0.625	15	0.27
1.25	15	0.22
2.5	15	0.28

Supplementary Fig. 4. Synergistic killing effect of P5091 and ABT263 on SnCs. (**a-c**) Comparison of mitochondria content and expression of mitochondrial pro- and anti-apoptotic proteins in Non-SnC and IR-SnC WI-38 cells. **(a)** Representative images of mitochondria and nucleus staining of non-SnC and IR-SnC WI-38 cells. Mitochondria and nucleus were stained by MitoTracker Red CMXRos (red) and DAPI (blue), respectively. Expression of the BCL-2 family proteins in Non-SnC, IR-SnC and REP-SnC WI-38 (**b**) and HUVEC cells (**c**) were detected by Western blotting. (**d**) Combination of P5091 and ABT263 synergistically kills SnCs. Viable IR-SnC WI-38 cells were determined 72 h after they were incubated with indicated concentrations of P5091 and/or ABT263. Percentages of viable cells are presented as mean ± SEM (n = 3) in the upper panels and the values of the combination index (CI) for each combination treatment are presented in the lower panels (CI < 1 indicating synergy).

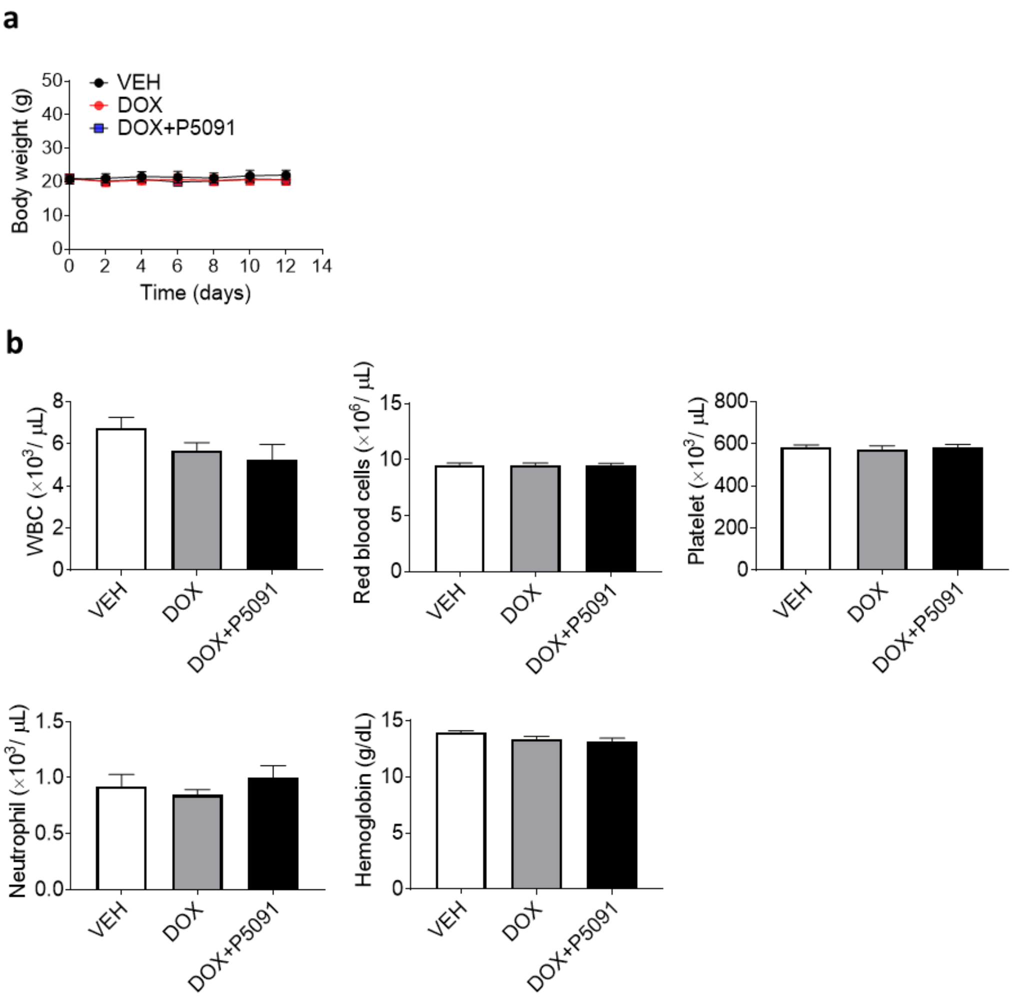

Supplementary Fig. 5. Treatment with P5091 does not cause observable toxicity in mice. (**a**) No body weight changes in mice after P5091 treatment for 12 days as shown in Fig. 4a. (**b**) P5091 treatment has no effect on various blood cell counts in mice. Levels of white blood cells (WBC), red blood cells, platelets, neutrophil, and hemoglobin in mice after receiving treatment with vehicle (VEH), doxorubicin (DOX), and DOX plus P5091 as shown in Fig. 4a. All the data are presented as mean ± SEM (n = 10).

Supplementary Table 1. Reagents and supplies used in *in vitro* and in *vivo* experiments

Name	Cat#	Vendor
DMEM	12430054	Thermo Fisher Scientific, Waltham, MA, USA
FBS	S11150H	Atlanta Biologicals, Flowery Branch, GA, USA
Penicillin-streptomycin	15140122	Thermo Fisher Scientific, Waltham, MA, USA
Renal epithelial cell medium	PCS-400-030	ATCC, Manassas, VA, USA
Renal epithelial cell growth kit	PCS-400-040	ATCC, Manassas, VA, USA
Fibroblast basal medium	PCS-201-030	ATCC, Manassas, VA, USA
Fibroblast growth kit	PCS-201-041	ATCC, Manassas, VA, USA
EGM-2 BulletKit	CC-3162	Lonza, Basel, Switzerland
Lipofectamine 2000 reagent	11668019	Life Technologies, Carlsbad, CA, USA
Polybrene	TR-1003-G	Sigma-Aldrich, St. Louis, MO, USA
Lipofectamine RNAiMax	13778075	Thermo Fisher Scientific, Waltham, MA, USA
Opti-MEM Medium	31985070	Thermo Fisher Scientific, Waltham, MA, USA
CellTiter-Glo Luminescent Cell Viability Assay kit	G7570	Promega, Madison, WI, USA
Q-VD-Oph	A1901	APExBIO, Houston, TX, USA
Alexa Fluor 647-Annexin V	640912	BioLegend, San Diego, CA, USA
Propidium iodide	P4170	Sigma-Aldrich, St. Louis, MO, USA
RIPA buffer	BP-115DG	Boston BioProducts, Ashland, MA, USA
Protease Inhibitor Cocktail	P8340	Sigma-Aldrich, St. Louis, MO, USA
Phosphatase Inhibitor Cocktail	P0044	Sigma-Aldrich, St. Louis, MO, USA
Pierce BCA protein Assay kit	23225	Thermo Fisher Scientific, Waltham, MA, USA
Mini-PROTEAN TGX	456-1094	Bio-Rad, Hercules, CA, USA
PVDF membrane	LC2002	Invitrolon, Life Technologies, Carlsbad, CA, USA
TBST	J77500	Affymetrix, Santan Clara, CA, USA
Non-fat dry milk	sc-2324	Santa Cruz Biotechnology, Dallas, TX, USA
Chemiluminescent HRP substrate	WBKLS0500	MilliporeSigma, Billerica, MA, USA
Cell Fractionation Kit	ab109719	Abcam, Cambridge, MA, USA
Pierce IP Lysis Buffer	87788	Thermo Fisher Scientific, Waltham, MA, USA
Pierce Protein A/G Magnetic Beads	88802	Thermo Fisher Scientific, Waltham, MA, USA
RNeasy Mini Kit	74106	Qiagen, Gaithersburg, MD, USA
cDNA Reverse Transcription Kit	4368813	Thermo Fisher Scientific, Waltham, MA, USA
TaqMan Fast Advanced Master Mix	4444965	Thermo Fisher Scientific, Waltham, MA, USA
Glass bottom microwell dishes	P35G-1.5-14-C	MatTek Corp., Ashland, MA, USA

MitoTracker Red CMXRos	M7512	Thermo Fisher Scientific, Waltham, MA, USA
4% paraformaldehyde	199431	Affymetrix, Santa Clara, CA, USA
Triton X-100	T8787	Sigma-Aldrich, St. Louis, MO, USA
BSA	BP9703	Fisher Scientific, Silver Spring, MD, USA
Anti-mouse IgG-Alexa Fluo488	A-11001	Thermo Fisher Scientific, Waltham, MA, USA
β-Galactosidase Staining Kit	9860	Cell signaling, Danvers, MA, USA
Click-iT Plus EdU Alexa Fluor 488 Imaging Kit	C10637	Thermo Fisher Scientific, Waltham, MA, USA
P5091	HY-15667	MedChem Express, Monmouth Junction, NJ, USA
P22077	HY-13865	MedChem Express, Monmouth Junction, NJ, USA

Supplementary Table 2. SgRNA sequence for p53 knockout by CRISPR/Cas9 genomic editing

**Name**	**Sequence or ID**	**Vendor**
TP53-sg 1 Forward	5’-CACCGACTTCCTGAAAACAACGTTC-3’	IDT, Coralville, IA, USA
TP53-sg 1 Reverse	5’-AAACGAACGTTGTTTTCAGGAAGTC-3’	IDT, Coralville, IA, USA
TP53-sg 2 Forward	5’- CACCGCCCCGGACGATATTGAACAA-3’	IDT, Coralville, IA, USA
TP53-sg 2 Reverse	5’-AAACTTGTTCAATATCGTCCGGGGC-3’	IDT, Coralville, IA, USA

Supplementary Table 3. Antibodies used for Western blotting analyses

**Name**	**Cat#**	**Vendor**
USP7	4833S	Cell signaling, Danvers, MA, USA
MDM2	86934S	Cell signaling, Danvers, MA, USA
p53	9282S	Cell signaling, Danvers, MA, USA
Phosphorylated p53	2521S	Cell signaling, Danvers, MA, USA
Cleaved PAPR	9541S	Cell signaling, Danvers, MA, USA
PAPR	9532S	Cell signaling, Danvers, MA, USA
P21	2947S	Cell signaling, Danvers, MA, USA
FAS	4233S	Cell signaling, Danvers, MA, USA
PUMA	4976S	Cell signaling, Danvers, MA, USA
BAX	2772S	Cell signaling, Danvers, MA, USA
BAK	12105S	Cell signaling, Danvers, MA, USA
NOXA	14766S	Cell signaling, Danvers, MA, USA
β-actin	4970S	Cell signaling, Danvers, MA, USA
GAPDH	97166S	Cell signaling, Danvers, MA, USA
CoxIV	4850S	Cell signaling, Danvers, MA, USA
BCL-XL	2762S	Cell signaling, Danvers, MA, USA
BCL-2	2872S	Cell signaling, Danvers, MA, USA
MCL1	5453S	Cell signaling, Danvers, MA, USA
P53 (for IP)	sc-126X	Santa Cruz, Dallas, TX, USA
IgG	sc-2025	Santa Cruz, Dallas, TX, USA

Supplementary Table 4. Taqman gene expression probes used for qRT-PCR

**Gene**	**Cat#**	**ID**
Human *CDKN2A*	4331182	Hs00923894_m1
Human *CDKN1A*	4331182	Hs00355782_m1
Human *PMAIP1*	4331182	Hs00560402_m1
Human *MDM2*	4331182	Hs00242813_m1
Human *FAS*	4331182	Hs00163653_m1
Human *BBC3*	4331182	Hs00248075_m1
Human *GAPDH*	4351370	Hs02758991_g1
Mouse *Cdkn2a*	4351370	Mm00494449_m1
Mouse *Hprt*	4351370	Mm01545399_m1
Mouse *Il1a*	4351370	Mm99999060_ m1
Mouse *Il1b*	4351370	Mm00434228_m1
Mouse *Il6*	4351370	Mm00446190_m1
Mouse *Tnfsf11*	4351370	Mm00441908_m1

Footnotes: All probes were purchased from Thermo Fisher Scientific, Waltham, MA, USA.
